# Welcome to *Gels*—An Interdisciplinary Open Access Journal for a Growing Scientific Community

**DOI:** 10.3390/gels1010001

**Published:** 2014-09-22

**Authors:** David Díaz Díaz

**Affiliations:** 1 Institut für Organische Chemie (CH 23.2.80), Fakultät für Chemie und Pharmazie, Universität Regensburg, Universitätsstr. 31, D-93053 Regensburg, Germany; David.Diaz@chemie.uni-regensburg.de; Tel.: +49-941-943-4373; 2 IQAC-CSIC, Jordi Girona 18-26, 08034 Barcelona, Spain

The ability of natural systems to change functions in response to altered environmental conditions has inspired scientists during the last decades to fabricate smart materials that respond to different stimuli (e.g., temperature, ionic strength, light, mechanical stress, electromagnetic radiation, mechanical stress, chemical additives). These responses are typically manifested as remarkable changes from the molecular to the macroscopic level. Among many kinds of stimuli-responsive materials, gels have been recognized as versatile functional materials for numerous exciting applications, in areas such as personal care, medicine, food, agriculture, bioengineering, art restoration, and electronic devices. Despite different forms of gels that are already well-integrated in everyday life, the development of new gels and their applications is growing at extraordinary pace, stimulating even the naissance of new paradigms that challenge established knowledge. Consider, for example, the concept of gel-like nature of the cytoplasm described by Gerald H. Pollack, which has confronted the wisdom on how cells work, and turned into a persuasive new foundation for cell biology.

When I first introduce gels to my undergraduate and graduate students, including practical examples, they feel immediately interested and fascinated by how they are formed, their dynamic nature (in contrast to the popular perception) and their widespread presence in our lives (including in our own body!). In fact, for many years I have found myself completely captivated by gels and their amazing properties! Gel materials are expected to form part of prominent solutions to major problems, such as limited resources, and safeguarding human health and the environment.

A quick look at the constantly increasing literature dealing with gels demonstrates that more and more researchers worldwide are becoming engaged in this fascinating topic. Thus, we have envisioned the convenience of a new journal devoted to these materials that could serve as a reference, by bridging the gap and promoting fruitful interactions between very different fields that, in one way or another, are beneficiaries of the properties of gel materials. 

For this journal, a broad range of gels will be considered. Articles covering in detail preparation methods, characterization, functionalities, mechanistic studies, and applications of either chemical or physical gels made from low molecular weight compounds or polymers—including, for example, organogels, hydrogels, aqueous gels, ionic gels, organic-inorganic hybrid gels—are within the scope of the journal. Theoretical studies with explicit solvent molecules, and studies focused on the relationship between solvent properties and gelation ability are also becoming increasingly important in the field, as they may contribute to a better understanding of gelation mechanisms. Hence, contributions of this nature will be also highly appreciated.

The publisher of *Gels* is committed to rapid dissemination of knowledge through an exemplar model for high quality publications under the open access format. While other publishers require access fees that are continuously increasing and, in many cases, very high costs for covering color images, MDPI charges only moderate article processing fees without institutional subscriptions. In this way, the journal aims to sustain a quick, yet rigorous peer-review process and rapid publication once a contribution has been accepted, which will be freely available to everyone in the world. Nevertheless, contributions published in the first two years of *Gels* will be exempt from article processing fees. This publishing model is also expected to encourage the research of countries that have not historically been very active in the field.

During the launch of the journal we will publish a series of special issues, based on areas of expansion and the latest advances in the field. One of the main aims of these issues is to provide a forum for researchers from different disciplines to learn and identify the major challenges in the field of gels. The broad scope of the journal, in terms of different kinds of gels, is expected to stimulate new discoveries by successful implementation of breakthrough concepts from one field in another. Within this context, we envision the first series of topical issues covering gels with self-healing properties; applications of gels in biomedicine, catalysis, sensors, environmental remediation, and energy; gelation of fluids at room temperature; gels as selective micro- or nanoreactors; elucidation of gelation mechanisms; and computational modeling of gel systems. Proposals for other topics that you think should be explored in a special issue are most welcome, as are appropriate guest editors.

On behalf of our Editorial Board, I cordially invite you to submit your articles to *Gels*, as well as your comments and ideas that can contribute to make this journal a prominent and unique platform for scientific discussion and rapid dissemination of knowledge in the fast-growing and interdisciplinary field of Functional *Gels*.


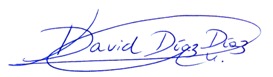

David Díaz Díaz
Editor-in-Chief of *Gels*

